# An Indirect Inguinal Hernia in a Neonate Containing the Uterus, Ovary, and Fallopian Tube: A Report of a Rare Case and a Literature Review

**DOI:** 10.7759/cureus.65440

**Published:** 2024-07-26

**Authors:** Satyanarayana Kummari, Sairam Subburam, Sree Raksha Chokkalingam

**Affiliations:** 1 Radiology, All India Institute of Medical Sciences, Nagpur, Nagpur, IND; 2 General Practice, Government Medical College, Omandurar Government Estate, Chennai, IND; 3 Medicine, Government Medical College, Omandurar Government Estate, Chennai, IND

**Keywords:** high-resolution ultrasonography, uterus and ovary, preterm neonate, congenital inguinal hernia, indirect inguinal hernia

## Abstract

An indirect inguinal hernia is a commonly seen congenital condition that can impact infants within their first year of life. An inguinal hernia arises when a portion of the intestines, omentum, or reproductive structures herniate into the scrotal sac or labia through the patent processus vaginalis. These hernias occur more frequently in preterm newborns. The contents of a hernia often consist of the small bowel, colon, omentum, and either the ovary or testicle. The occurrence of a uterus in a herniated sac is uncommon. The presence of a uterus, ovary, and fallopian tube is exceptionally rare, with only a few cases reported in the literature.

We present a unique case of a 10-day-old female neonate who was delivered at 37 weeks of gestation and brought to the Paediatric Outpatient Department with swelling in the left inguinal region that had been present for the past five days. During the clinical examination, an irreducible mass was found in the left inguinal region. The ultrasound scan showed the uterus, ovary, fallopian tube, and minimal free fluid in the herniated sac. Colour Doppler evaluation of the uterus and ovaries revealed good vascularity. A diagnosis of a left inguinal hernia containing the uterus, left ovary, and fallopian tube, with no signs of ovarian torsion, was established. We performed a surgical procedure in which the likelihood of adhesions was taken into consideration, and the organs were removed from the hernial sac. We conducted the reintegration of the organs back into the pelvis, ligation of the high sac, and further repair of the internal inguinal ring to prevent the recurrence of the hernia. The surgical procedure was successful, and the postoperative period was without any complications. After the surgery, the patient was advised to have clinical and radiological follow-up for a period of one year. We recommend that a high-resolution ultrasound (HRUS) scan be routinely performed in neonates with an asymptomatic or symptomatic palpable mass in the inguinal region for early diagnosis and characterization of the herniated structures, as well as to assess their viability.

## Introduction

Infants and children can be affected by several types of congenital abnormalities. An indirect inguinal hernia is a commonly seen congenital condition that can impact infants within their first year of life, with an incidence ranging from 0.8% to 4% [1-3]. The development of an inguinal hernia is a consequence of inadequate closure of the inguinal canal. It is possible for the herniated sac to contain various organs, such as the small intestine, large intestine, omentum, free fluid, testicles, ovaries, fallopian tubes, uterus, and urinary bladder, depending on the gender of the affected patient [1,2]. In about 15-20% of female infants, the herniated sac contains the ovary and/or fallopian tube. However, it is worth noting that only a few cases contain the ovaries, fallopian tubes, and uterus within the hernial sac [1,2]. Only a small number of these hernias regress spontaneously [4]. On the other hand, if the herniated sac contains any portions of the intestines or ovaries, the likelihood of spontaneous regression decreases while the possibility of incarceration increases [5]. Therefore, it is necessary to diagnose and intervene early to prevent permanent damage to the contents of the hernia.

High-resolution ultrasound (HRUS) scan with colour Doppler is the primary and highly effective diagnostic tool for assessing inguinal lesions [6,7]. The objective of the present case report is to present an exceptionally uncommon case of a 10-day-old female infant with a left inguinal hernia that contains the uterus, ovary, and fallopian tube. Furthermore, it aims to demonstrate the significance of ultrasonography in diagnosing congenital inguinal hernia containing the uterus, ovary, and fallopian tube during infancy and raise awareness among sonographers about its manifestation and presentation.

## Case presentation

**Figure 1:**
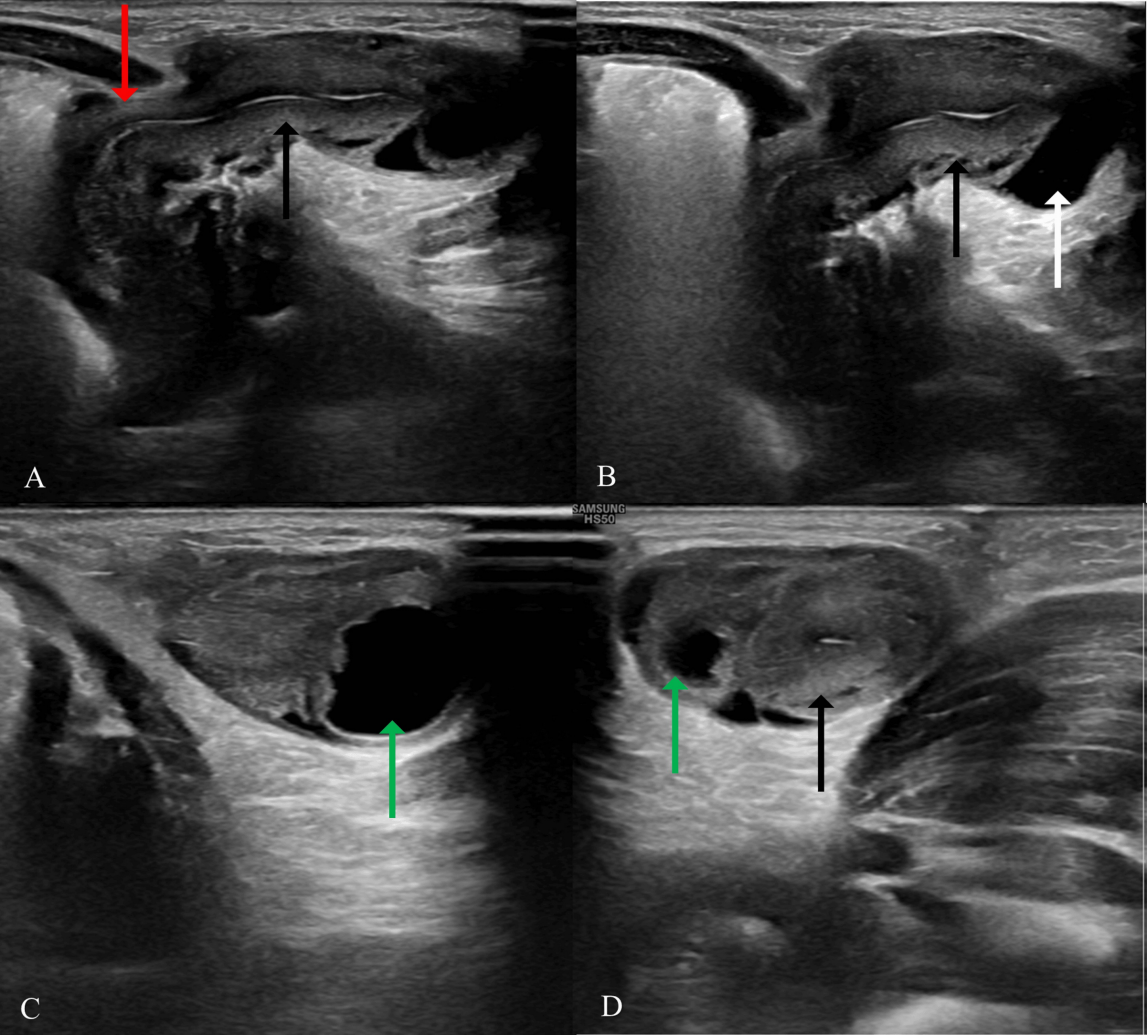
Ultrasonography images of the left inguinal region (A)-(B) Sagittal images show the uterus (black arrows), mild fluid (white arrow) and defect of the hernia (red arrow); (C)-(D) Axial images show the uterus (black arrows) and ovary (green arrow)

A 10-day-old female neonate who was delivered at 37 weeks of gestation and weighed 2800 g at birth was brought to the Paediatric Outpatient Department with a swelling in her left inguinal region that had been present for the past five days. There were no previous records of unusual events associated with the vaginal delivery, and there were no signs of irritation, redness, pain, vomiting, or evidence of inflammation in the left inguinal region. During the clinical examination, a swelling was found in the left inguinal region that could not be reduced, and the skin covering it appeared normal. An ultrasound scan of the left inguinal region was recommended to rule out unusual structures, such as the uterus and ovaries. The scanning approach utilized HRUS with a linear probe to evaluate the site of the swelling. The scanning planes varied based on the specific location of the swelling. The HRUS scan revealed a hypoechoic structure with an internal echogenic strip that extended into the pelvic cavity through a defect in the lower abdominal wall (uterus). Additionally, an anechoic structure (fallopian tube), a hypoechoic structure with anechoic areas within it (ovary), and mild free fluid were also observed. In terms of measurement, the neck of the hernia was around 9 mm. It was an irreducible hernia. The uterus demonstrated normal size, shape, and echogenicity. The left ovary exhibited a normal size, shape, and echotexture and contained a few follicles (Figure 1). 

The right ovary was of normal size, shape, and echotexture and was located in the pelvis. The colour Doppler assessment of the uterus and ovaries demonstrated adequate blood flow, indicating the presence of normal uterine and ovarian tissue. It was determined that the patient had a left inguinal hernia, with the uterus, left ovary, and fallopian tube as its contents; however, there were no signs of ovarian torsion. The patient was sent to the paediatric surgery unit for further treatment. In infants, surgery (including correction, reduction, and ligation) is typically the preferred treatment option. We performed a surgical procedure in which the likelihood of adhesions was taken into consideration, and the organs were removed from the hernial sac. We conducted the reintegration of the organs back into the pelvis, ligated the high sac, and performed further repair of the internal inguinal ring to prevent the recurrence of the hernia. The surgical procedure was successful, and the postoperative period was without any complications. After the surgery, the patient was advised to have clinical and radiological follow-up for a period of one year.

## Discussion

Infants and children can be affected by several types of congenital abnormalities. An indirect inguinal hernia is a commonly seen congenital condition that can impact infants within their first year of life. It is more common in pre-term babies [1-3].

Approximately 33% of children experience the development of hernias before they reach six months of age; furthermore, the majority of hernias occur in boys, with a boys-to-girls ratio of 6:1. The development of an inguinal hernia is a consequence of inadequate closure of the inguinal canal. The processus vaginalis develops as a protrusion of the parietal peritoneum approximately six months after conception. The processus vaginalis is accompanied by either the testis or the round ligament of the uterus, depending on the gender of the foetus. It traverses the inguinal canal toward the scrotal sac in males or the labia majora in females. The canal of Nuck, which is the female equivalent of the processus vaginalis, is typically smaller in size and often disappears by the eighth month of gestation. Consequently, the likelihood of developing an inguinal hernia increases when a baby is delivered before the closure of this canal [8-10].

Inguinal hernias can potentially contain many organs and structures, such as the small intestine, large intestine, omentum, free fluid, testicles, ovaries, fallopian tubes, uterus, and urinary bladder, depending on the gender of the affected patient [1,11,12]. Children diagnosed with Complete Androgen Insensitivity Syndrome (CAIS), formerly known as Testicular Feminization Syndrome, have female external genitalia and endocrine function but possess undescended testes within the abdominal cavity or inguinal canal instead of ovaries. Therefore, with regard to CAIS, approximately 1.6% of children diagnosed with an inguinal hernia may have their testicles located in the hernial sac, especially if the condition affects both sides. Incarceration is a serious consequence of inguinal hernias in children and adolescents, occurring with a frequency of 31%, as reported in the literature [13]. The organs most frequently affected by incarceration include the intestines, ovaries, and fallopian tubes. While certain hernias may naturally resolve on their own, this is uncommon if the hernia involves the ovary. Additionally, compared to a hernia involving the bowel, there is a significantly higher chance of incarceration. An incarcerated hernia can rapidly develop into strangulation, which is a condition where the blood supply to the incarcerated contents is compromised, leading to infarction of the contents. Female infants with incarcerated ovaries are similarly at risk for torsion. Typically, uncomplicated inguinal hernias are characterized by episodic swelling in the inguinal area. In most cases, the swelling is painless and can be easily reduced by applying a small amount of pressure. A strangulated hernia typically manifests as a rigid, sensitive, irreducible mass in the inguinal area [14].

HRUS is a readily available and precise imaging tool. HRUS with colour Doppler is the preferred imaging method for evaluating and determining the status of herniated structures. Ultrasonography is used to distinguish the inguinal hernia from various other pathologies, such as hydrocele of the canal of Nuck, lymphadenopathy, Bartholin gland cyst, infection or abscess, inguinal gonads, and endometriosis. It is also used to examine the possibility of involvement on the other side. The ultrasound scan should be done on both inguinal regions, as it has been observed that a clinically undetectable hernia on the opposite side can be detected in 88% of patients [14,15].

During the ultrasound examination, the bowel loops in the hernial sac appear as fluid or air-filled tubular structures. The uterus shows its characteristic appearance as a tubular hypoechoic structure with a central hyperechoic endometrial line, and the ovaries appear as hypoechoic structures with several follicles within. Ovarian torsion is characterized by an enlarged ovary that appears as a mass with heterogeneous echogenicity and numerous peripheral cysts, without any blood flow in the ovary. For additional confirmation, a colour Doppler ultrasound can be used to assess the blood vessels in the ovarian pedicle and ascertain whether or not ischemia has occurred in the torsioned and herniated ovaries [16-19].

Only a small number of indirect inguinal hernias regress spontaneously [4]. On the other hand, if the herniated sac contains any portions of the intestines or ovaries, the likelihood of spontaneous regression decreases, while the possibility of incarceration increases [5]. Therefore, it is necessary to diagnose and intervene early to prevent irreversible damage to the herniated structures. In infants, surgery is typically the preferred treatment option. The surgical procedure includes the reintegration of the organs back into the pelvis, ligation of the high sac, and further repair of the internal inguinal ring to prevent the recurrence of the hernia [4,5,19].

## Conclusions

Incarceration is a serious consequence of inguinal hernias in infants, children, and adolescents. The organs most frequently affected by incarceration include the intestines, ovaries, and fallopian tubes. Therefore, it is necessary to diagnose and intervene early to prevent permanent damage to the contents of the hernia. HRUS with colour Doppler is the primary and most effective diagnostic tool for assessing inguinal lesions. We recommend that HRUS be routinely performed in neonates with asymptomatic or symptomatic palpable masses in the inguinal region for early diagnosis and characterization of the herniated structures, as well as their viability, to prevent irreversible damage to the herniated structures.

## References

[1] Karadeniz Cerit K, Ergelen R, Colak E, Dagli TE (2015). Inguinal hernia containing uterus, fallopian tube, and ovary in a premature newborn. Case Rep Pediatr.

[2] Comella BP, Fortes PO, Salvador RL (2019). Inguinal hernia containing uterus in a newborn: what to do?. Pediatr Neonatol.

[3] George EK, Oudesluys-Murphy AM, Madern GC, Cleyndert P, Blomjous JG (2000). Inguinal hernias containing the uterus, fallopian tube, and ovary in premature female infants. J Pediatr.

[4] Huang CS, Luo CC, Chao HC, Chu SM, Yu YJ, Yen JB (2003). The presentation of asymptomatic palpable movable mass in female inguinal hernia. Eur J Pediatr.

[5] Cascini V, Lisi G, Di Renzo D, Pappalepore N, Lelli Chiesa P (2013). Irreducible indirect inguinal hernia containing uterus and bilateral adnexa in a premature female infant: report of an exceptional case and review of the literature. J Pediatr Surg.

[6] Artas H, Gurbuzer N (2012). Inguinal hernia containing both ovaries and the uterus in an infant. J Ultrasound Med.

[7] Muthiyal S, Kini V, Kounsal A, Ibrahim AA (2016). Rarity in conspicuity-ultrasound diagnosis of sliding left inguinal hernia through canal of Nuck with uterus, fallopian tubes and ovaries. Eur J Radiol Open.

[8] Khanna PC, Ponsky T, Zagol B, Lukish JR, Markle BM (2007). Sonographic appearance of canal of Nuck hydrocele. Pediatr Radiol.

[9] Shadbolt CL, Heinze SB, Dietrich RB (2001). Imaging of groin masses: inguinal anatomy and pathologic conditions revisited. Radiographics.

[10] Merriman TE, Auldist AW (2000). Ovarian torsion in inguinal hernias. Pediatr Surg Int.

[11] Ziegler MM (1994). Diagnosis of inguinal hernia and hydrocele. Pediatr Rev.

[12] Ming YC, Luo CC, Chao HC, Chu SM (2011). Inguinal hernia containing uterus and uterine adnexa in female infants: report of two cases. Pediatr Neonatol.

[13] Shalev J, Mashiach R, Bar-Hava I, Girtler O, Bar J, Dicker D, Meizner I (2001). Subtorsion of the ovary: sonographic features and clinical management. J Ultrasound Med.

[14] Aso C, Enríquez G, Fité M, Torán N, Piró C, Piqueras J, Lucaya J (2005). Gray-scale and color Doppler sonography of scrotal disorders in children: an update. Radiographics.

[15] Moss RL, Hatch EI (1991). Inguinal hernia repair in early infancy. Am J Surg.

[16] Aydin R, Polat AV, Ozaydin I, Aydin G (2013). Gray-scale and color Doppler ultrasound imaging findings of an ovarian inguinal hernia and torsion of the herniated ovary: a case report. Pediatr Emerg Care.

[17] Ogata M, Mateer JR, Condon RE (1996). Prospective evaluation of abdominal sonography for the diagnosis of bowel obstruction. Ann Surg.

[18] Blaivas M (2002). Ultrasound-guided reduction of a Spigelian hernia in a difficult case: an unusual use of bedside emergency ultrasonography. Am J Emerg Med.

[19] Ogata M, Imai S, Hosotani R, Aoyama H, Hayashi M, Ishikawa T (1994). Abdominal ultrasonography for the diagnosis of strangulation in small bowel obstruction. Br J Surg.

